# Pharmacoepidemiology of antipsychotic utilization among children and adolescents in Montevideo between 2018 and 2022

**DOI:** 10.3389/fphar.2026.1778138

**Published:** 2026-04-28

**Authors:** Santiago Cabral, Valentina Catenaccio, Guadalupe Herrera, Susana Grunbaum, Noelia Speranza

**Affiliations:** 1 Unidad Académica de Farmacología y Terapéutica, Facultad de Medicina, Universidad de la República, Montevideo, Uruguay; 2 Unidad Académica de Medicina Preventiva y Social, Facultad de Medicina, Universidad de la República, Montevideo, Uruguay; 3 United Nations International Children’s Emergency Fund (UNICEF), Montevideo, Uruguay

**Keywords:** adolescent, antipsychotic agents, child, mental health, pharmacoepidemiology

## Abstract

**Background:**

The prescription of antipsychotics in the pediatric population has increased worldwide, especially of second generation antipsychotics. Prescription patterns are not homogeneous across populations. Studies demonstrate differences according to sex, age and socioeconomic level. In Uruguay, the available evidence is limited and comes from small, single-center studies and does not provide information on changes over time.

**Purpose:**

describe the use of antipsychotics in 1- to 19-year-old children and adolescents in Montevideo between 2018 and 2022, considering differences according to socioeconomic level.

**Study design:**

A descriptive observational study of medication use was conducted.

**Methods:**

It included children and adolescent users of antipsychotics treated in eight healthcare centers in Montevideo (one public, five collective healthcare institutions, and two private insurance schemes), which together provide coverage to approximately 230,000 children and adolescents in total. Pharmacy dispensing data were analyzed between 1/1/2018 and 12/31/2022. The primary endpoint was the defined daily dose (DDD) per 1,000 inhabitants/day (DDD per 1,000 inhabitants/day), calculated globally and by drug. Temporal variation was evaluated in three periods: pre-pandemic (2018–2019), start of the pandemic (2020) and post-pandemic (2021–2022). Descriptive statistics were used.

**Findings:**

The average DDD per 1,000 inhabitants/day in the period was 11.4, with a predominance of risperidone and aripiprazole. The highest utilization was observed among adolescents aged 15–19 years, followed by the group aged 5–14 years, and was higher in males. Although the use of antipsychotics increased over time in all socioeconomic levels, it was higher for providers of care at the low socioeconomic level and lower for providers of care at the high socioeconomic level.

**Conclusion:**

This is the first study in Uruguay that describes the use of antipsychotic agents in children and adolescents by socioeconomic level; it showed a steady increase in their use.

## 1 Introduction

Mental health disorders in childhood and adolescence reach an estimated prevalence of approximately 20% worldwide (National Institute of Human Rights and Ombudsman’s Office, 2019; Orueta Sánchez et al., 2011). In Uruguay, population-based studies have reported prevalences exceeding 20% among children living in Montevideo (Viola et al., 2008).

Mental health problems in pediatric populations are not evenly distributed, but are strongly shaped by social determinants of health, with consistently higher prevalence observed in contexts of socioeconomic vulnerability (Observatory of the Uruguayan health system, 2025).

From both public health and human rights perspectives, mental healthcare should be grounded in comprehensive, and integrated models of care, ensuring equitable access to both pharmacological and non-pharmacological interventions (National Mental Health Program and General Directorate of Health, 2011).

Within this framework, the use of psychotropic medications (particularly antipsychotics) in pediatric populations has increased (Alessi-Severini et al., 2012; Radojčić et al., 2023). Antipsychotics are currently prescribed for a wide range of psychiatric and behavioral indications, including irritability, aggression, mood disorders, autism spectrum-related symptoms, and attention-deficit/hyperactivity disorder, between 70% and 85% of prescriptions for off-label indications (Radojčić et al., 2023; Sohn et al., 2016). In Uruguay, the technical data sheets for medications, approved by the Ministry of Health, are not available, so it is only possible to estimate on-label uses similar to those reported in other countries’ labels like the United States (Valtuille et al., 2024).

Antipsychotics have adverse effects that can be explained by their mechanism of action. Typical antipsychotics, with greater dopamine D2 antagonism, carry a risk of causing parkinsonism and hyperprolactinemia; in children and adolescents, acute dystonia presents a greater risk. Atypical antipsychotics, with lesser dopamine D2 antagonism and 5-HT2A serotonin antagonism, have a lower incidence of these risks, but are associated with metabolic disturbances such as weight gain, hyperglycemia, and dyslipidemia. Antimuscarinic adverse effects, orthostatic hypotension, and an increased risk of sedation are common to both typical and atypical antipsychotics (Meyer et al., 2022). In Uruguay, there are national recommendations for monitoring children and adolescents using these medications, which include a schedule of medical visits with anthropometric and metabolic blood biochemistry assessments (Assandri et al., 2020). Despite this, few children are properly monitored (Cabral et al., 2025a).

International evidence consistently identifies risperidone, quetiapine, and aripiprazole as the most commonly prescribed agents in children and adolescents. The increased use of these drugs is causing concern in the medical community due to the exposure of children and adolescents to these neurological and metabolic risks (Mastroianni et al., 2017; Dörks et al., 2023; Bushnell et al., 2021; Ismael et al., 2010).

Prescription patterns are not homogeneous across populations. Studies demonstrate differences according to sex, with male children and adolescents showing substantially higher rates of antipsychotic exposure (Alessi-Severini et al., 2012; Olfson et al., 2006). Likewise, socioeconomic gradients have been described, with higher prescription prevalence among individuals receiving care in public health systems compared to private providers, approximately 2–5 times more, suggesting the influence of structural and institutional determinants on pharmacological treatment patterns (Alessi-Severini et al., 2012; Patel et al., 2005).

Between 2018 and 2022, mental healthcare delivery occurred within the broader context of the COVID-19 pandemic, which profoundly disrupted health systems. In Uruguay, the early phase of the pandemic was characterized by reduced mobility, decreased in-person consultations, and limited access to mental health services, followed by the rapid implementation of telemedicine and electronic prescription systems (Ferre et al., 2021). It is estimated that the pandemic increased mental health problems in the population, including children and adolescents (Honorary Scientific Advisory Group and Ministry of Public Health, 2021).

Given the increasing use of antipsychotics in pediatric populations, the known influence of sex and socioeconomic determinants on prescribing practices, and the structural transformations in healthcare delivery during the pandemic period, there is a clear need for local, population-based descriptive evidence.

The aim of this study was to describe the use of antipsychotics among children and adolescents aged 1–19 years in Montevideo between 1 January 2018 and 31 December 2022, according to age, sex, and socioeconomic level.

## 2 Materials and methods

### 2.1 Study design

The cross-sectional observational descriptive study of medication utilization conducted included data on pharmacy dispensing of any antipsychotic drug to children and adolescents aged 1–19 years, in Montevideo, as prescribed by public and private healthcare providers from 1 January 2018 to 31 December 2022. Given the increasing use of antipsychotics at young ages, with previous studies confirming this fact, infants were included to document this finding (Cabral et al., 2025b).

### 2.2 Data source

The institutions from Montevideo invited to participate were selected on the basis of convenience. Since 2007, Uruguayan healthcare has been ruled by an integrated health system (Sistema Nacional Integrado de Salud, SNIS), which is binding to both public and private providers (Pan American Health Organization, 2021; Lausarot et al., 2010). The latter include Collective Medical Care Institutions (IAMC) and private health insurance schemes. All of them were included as potential participants in the study.

The type of healthcare provider in charge was considered a good estimate of the socioeconomic level (SEL) of the population to be included. According to the characterization of SNIS users prepared by the Ministry of Health (MSP), the public subsector has the highest proportion of users in the first and second quintile of family income, and a share of both unemployed people and people on unemployment subsidies. By way of contrast, more than 90% of the beneficiaries of private insurance schemes are between the fourth and fifth income quintile (Ministry of Health, 2018; Ministry of Health, 2021). Based on this characterization, it was assumed that the public and private (mutual and insurance scheme) providers can be deemed low, middle, and high SEL, respectively, as has been done in other Uruguayan studies (Briozzo et al., 2025).

When a person picks up medication at their healthcare institution’s pharmacy, this action is recorded in each pharmacy’s database, which collects the person’s demographic data and information about the dispensed medication, including the amount of active ingredient provided. These databases are heterogeneous among the providers and the name off the pharmacy management software used was not collected.

In Uruguay, there are no national databases that compile this information, and the Ministry of Health does not collect this data.

Pharmacies at participating institutions were asked to provide outpatient dispensing data for all antipsychotics available in their institutional formulary, specifically for children and adolescents during the period of interest. The technical director of each healthcare institution authorized the release of a copy of the database containing the requested information. The datasets were then reviewed and the variables were standardized and harmonized across institutions to ensure consistency in coding and structure (for some institutions, the authors had to convert the number of boxes dispensed and milligrams per tablet into total milligrams dispensed).

Subsequently, these databases were merged by the authors into a single spreadsheet for analysis. All information obtained was anonymized.

All antipsychotics available in the country were included: chlorpromazine, levomepromazine, chlorprothixene, haloperidol, periciazine, and thioridazine (first-generation antipsychotics); and aripiprazole, clozapine, olanzapine, quetiapine, risperidone, sulpiride, tiapride, and lurasidone (second-generation antipsychotics). Only oral formulations were included; preparations for parenteral administration (intramuscular/intravenous) were excluded.

Dispensing was assumed to reflect actual drug consumption by individuals. It was also assumed that all data provided were accurate, as they were obtained through automated registration processes. However, since it was not possible to determine whether this process was performed automatically through the download of a file or manually, we made additional efforts to assess the accuracy and quality of the data. No inconsistencies were detected through manual review of the spreadsheet, and no anomalies were found during data analysis.

### 2.3 Evaluation indicators and statistical analysis

The primary endpoint used to estimate utilization was the variable recommended by the World Health Organization (WHO), the defined daily dose (DDD) per 1,000 inhabitants/day (DDD per 1,000 inhabitants/day) (World Health Organization, 2025; Figueiras and Antoni, 2003). This is defined as an international technical unit for measuring drug utilization in a population. The DDD, which corresponds to the average daily maintenance dose of a drug in its main indication in adults, is used for the calculation. There is no definition of DDD for pediatric ages and there is no consensus of which standard doses use for children in drug utilization studies. This is because the doses vary widely depending on the weight of the individuals, as well as the indication of the drug. The decision was made to use the DDD for adults, as suggested by the WHO, in order to use a standardized measure for comparisons, knowing the limitations it would entail when analyzing the results (World Health Organization). According to this assumption, the result of this measure in this study could be interpreted as the number of children and adolescents out of every 1000 inhabitants under 19 years of age in Montevideo that took a defined daily adult dose of an antipsychotic daily.

Added to that, the main indication for antipsychotics in adults does not match the indications approved for children and again, many are prescribed off label. This data could not be collected using this methodology since pharmacy software does not necessarily require this data for dispensing medication and therefore it is not collected. The DDD per 1,000 inhabitants/day was calculated for each antipsychotic for each year of the study period. For the number of DDD, the sum of the milligrams of each antipsychotic dispensed by each institution in a year. To calculate the DDD per 1,000 inhabitants/day, the population was considered to be the sum of the users of the specific age range for each healthcare provider.

The DDD per 1,000 inhabitants/day was calculated for each individual antipsychotic, in each year of the selected period, for users 1–19 years of age by age groups, roughly as follows: preschool, school and adolescence would respectively correspond to 1–4 years, 5–14 years and 15–19 years, and by sex (male/female).

The formula used to calculate antipsychotic use in children and adolescents was:

$$DDD\;1,000\;inhabitants/day=\left(mg\;dispensed/DDD\;\left(mg\right)\;*\;1000\;inhabitants\right)/\left[N\;population\;*\;time\;\left(days\right)\right]$$

where:

mg = sum of milligrams of each of the antipsychotics dispensed per year.

DDD = defined daily dose according to WHO.

time = 365 days.

N population = total number of users in the age range considered in the institution.

A global DDD per 1,000 inhabitants/day for all antipsychotics was estimated by adding the DDD per 1,000 inhabitants/day of the total antipsychotics of the centers included in the study, for each year in the study period, by age and sex, for each center, according to the SEL they represent. The overall DDD per 1,000 inhabitants/day was represented as the average for the study period.

Temporal variations in the study period were described for three time periods: pre pandemic COVID-19 period (2018 and 2019), the start of the pandemic (2020) and final stage of the pandemic (2021 and 2022), both overall and by antipsychotic.

All the data was combined into a Microsoft Excel spreadsheet. Processing and analysis were performed manually using the previously presented mathematical equations. Through a cross-review process, each author performed a portion of the calculations, and their results were then reviewed by another author. Additionally, a colleague external to the study reviewed the calculations.

The unit of description was the healthcare institution-year. This ecological approach allows comparison of utilization patterns across providers representing different socioeconomic strata. Given the aggregated nature of the data and the descriptive aim of the study, no confidence intervals or hypothesis tests were calculated. The analysis was descriptive in nature. Temporal variations over the 5-year period were presented by calculating annual rates, although no specific statistics were used for further analysis. No inferential statistical tests were performed, as the objective of the study was to describe patterns of medication utilization rather than to test specific hypotheses.

The data were presented using descriptive statistics.

### Ethics statement

The protocol was registered with the Ministry of Health (Registration number: 7851394) and was approved by the Research Ethics Committee of the Medical School of the Universidad de la República (File number 070153–000046–23). It was also presented to and approved by the respective committees of the participating institutions, when available.

User anonymity was guaranteed by pooling the data to prevent the identification of participants, with the endorsement of the technical departments of each provider.

## 3 Results

Of the 8 healthcare providers that participated, one is from the public subsector (considered low SEL), five are from IAMC (considered medium SEL) and two have private insurance schemes (considered high SEL).

The average annual population of users under 19 years of age of these eight institutions was 230,000 children and adolescents, which represents 55% of the population of this age in Montevideo between the years 2018–2022, according to the data reported by the National Data System (National Information System SINADI and Ministry of Public Health, 2024).

### 3.1 Pharmacological analysis

The sum of the average DDD per 1,000 inhabitants/day of antipsychotics for the 5 years of study was 11.41. The most common antipsychotics were risperidone, aripiprazole, and quetiapine.

**FIGURE 1:**
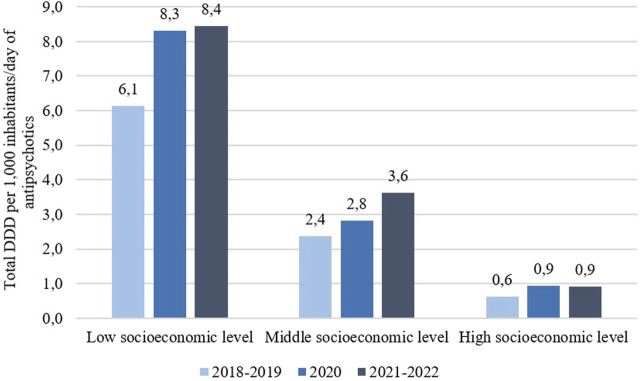
Changes over time in the total DDD per 1,000 inhabitants/day of antipsychotics in the period 2018–2022 in the three socioeconomic levels. Source: Prepared by the authors.

Between 2018 and 2022, there was an overall increase in the use of antipsychotic in all SELs. With respect to the DDD per 1,000 inhabitants/day values for these three antipsychotics, a regular pattern was observed across healthcare sectors. Higher DDD per 1,000 inhabitants/day values were found in institutions whose user populations have, on average, lower socioeconomic profiles, intermediate values in the medium group, and lower values in institutions serving populations with higher average socioeconomic levels. Figure 1 shows the changes over time in DDD per 1,000 inhabitants/day by type of provider. The DDDs per 1,000 inhabitants/day per drug, sex and age in the period 2018 to 2022 according to the SEL are presented as supplementary tables.

### 3.2 DDD per 1,000 inhabitants/day by age group

**FIGURE 2:**
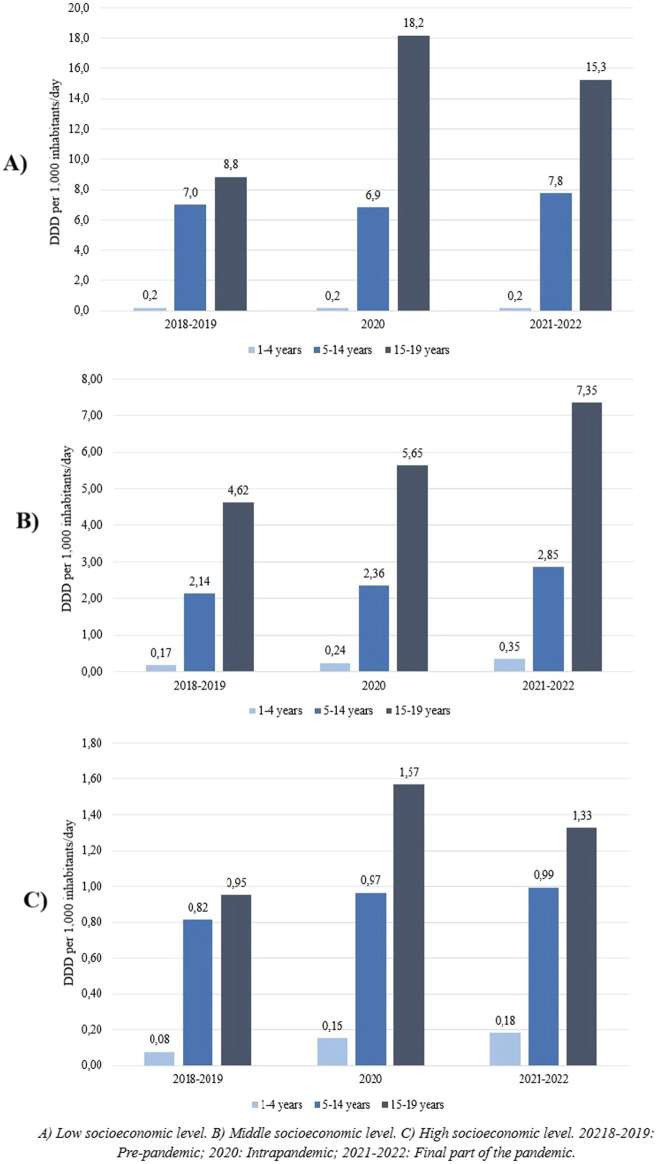
Changes over time in the DDD per 1,000 inhabitants/day by age range. **(A)** Low socioeconomic level. **(B)** Middle socioeconomic level. **(C)** High socioeconomic level. 20218–2019: Pre-pandemic; 2020: Intrapandemic; 2021–2022: Final part of the pandemic.

In the three SELs, antipsychotics are predominantly dispensed to adolescents (age group 15–19 years), followed by school children and adolescents (5–14 years), while prescriptions for preschoolers (1–4 years) are very low. In the public subsector and among the clients of private insurance there is an increase in DDD per 1,000 inhabitants/day in 2020 in the 15–19 age group; figures drop in the final period of the study (2021–2022) but remain higher than the baseline values (Figure 2).

### 3.3 DDD per 1,000 inhabitants/day by sex

**FIGURE 3:**
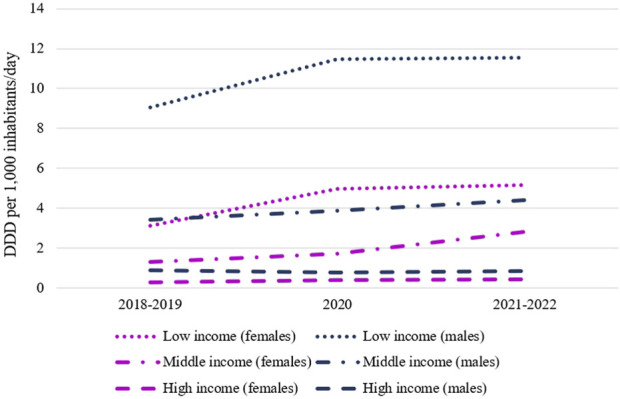
Changes over time in DDD per 1,000 inhabitants/day by sex and socioeconomic level. Source: Prepared by the authors.

Throughout the study period all healthcare providers dispensed more antipsychotic drugs to males in all age groups. Figure 3 shows the changes over time in DDD per 1,000 inhabitants/day by sex and SEL. The evaluation of the same data on a drug-by-drug basis showed that this trend was maintained.

### 3.4 DDD per 1,000 inhabitants/day by antipsychotics

Utilization of chlorpromazine, chlorprothixene, clozapine, lurasidone, tiapride, sulpiride, and periciazine was close to 0. No thioridazine dispensing was observed in any provider. Hence, in the analysis of the changes over time by drug, these drugs were not disaggregated, although they were included in the total estimates. Table 1 presents the estimation of total DDD per 1,000 inhabitants/day (between the periods 2018–2019 to 2021–2022) in the overall dispensing of antipsychotics and for each one, in the three health subsectors.

**TABLE 1:** DDD per 1,000 inhabitants/day per drug in the periods 2018/19 to 2021/22 in the three socioeconomic levels.

Active ingredient	Low income			Middle income			High income		
	2018–2019	2020	2021–2022	2018–2019	2020	2021–2022	2018–2019	2020	2021–2022
Risperidone	2,994	2,520	2,875	0,816	0,856	1,009	0,337	0,378	0,336
Aripiprazole	1,010	1,488	2,171	0,644	1,025	1,592	0,094	0,339	0,388
Quetiapine	0,850	0,915	1,001	0,430	0,429	0,477	0,087	0,100	0,114
Olanzapine	0,508	2,487	1,592	0,091	0,073	0,114	0,001	0,023	0,020
Haloperidol	0,337	0,429	0,322	0,113	0,082	0,098	0,001	0,004	0,006
Levomepromazine	0,313	0,369	0,373	0,139	0,160	0,131	0,019	0,016	0,020
All antipsychotics*	6,141	8,309	8,448	2,379	2,821	3,630	0,628	0,946	0,913

* Includes, in addition to the agents listed above, chlorpromazine, chlorprothixene, lurasidone, periciazine, sulpiride, clozapine and tiapride.

Early in the study (2018–2019) risperidone was the most commonly used antipsychotic, in the middle and high SEL healthcare providers, but by the end of the period it was positioned as the second most used antipsychotic after aripiprazole. Quetiapine was reported as the third most dispensed antipsychotic by the three types of providers.

With respect to the DDD per 1,000 inhabitants/day values of these three antipsychotics specifically, the utilization of these drugs showed regularity, with the lowest SEL presenting the highest DDD per 1,000 inhabitants/day values for each antipsychotic, intermediate values at the medium level and lower values at the high level.

## 4 Discussion

### 4.1 General findings

This is the first study in Uruguay that describes the utilization of antipsychotics in children and adolescents based on socioeconomic (SEL) differences, with a representativeness greater than 55% of the children and adolescents in Montevideo.

The increase in the use of antipsychotics in children and adolescents in Montevideo is also observed in other regions (Ismael et al., 2010; Menard et al., 2020). A study conducted in Germany that included 12 million children and adolescents (1–19 years) between 2011 and 2020 with at least one prescription of antipsychotics identified an increase in the prevalence of the use of these medications ranging from 3.16 to 3.65 per 1000 due to an increase in the use of both typical (1.16–1.35 per 1000) and atypical (2.35–2.75 per 1000) antipsychotics (Dörks et al., 2023). Also the highest dispensing of antipsychotics among adolescents has been observed in other countries (Dörks et al., 2023; Chavez et al., 2021). This could be explained by the higher prevalence of behavioral and emotional disturbances observed at these ages, which is accompanied by the use of off-label antipsychotics for these situations (Chen et al., 2021; World Health Organization, 2021). The use of antipsychotics in preschoolers aged 1–4 years in the study was low, close to zero, probably due to a lower prevalence of behavioral symptoms at these ages. The use of antipsychotics was found in children as young as 2 years of age. However, their use at such early ages is noteworthy, even more so considering the lower clinical evidence of efficacy and safety in this age group. This has already been confirmed in previous studies.

The use of antipsychotics was higher among males in all age groups, as has been reported in other countries (Alessi-Severini et al., 2012; Bushnell et al., 2021; Ismael et al., 2010; Olfson et al., 2006). Males tend to present the so-called “externalized symptoms” (irritability, hetero-aggressiveness), which are more striking than the “internalized symptoms” (withdrawal, sadness) more frequently observed in females; this may make them more prone to seek care and be prescribed treatment (Alarcón and Bárrig, 2015). In addition, it was found that the difference in use according to sex was maintained in all age ranges and across all SELs of the population studied. It is noteworthy that in the public sector the difference in use between sexes was much greater than in the other providers.

The highest utilization of antipsychotics was observed in the provider classified as serving populations with lower average SEL, whereas the lowest utilization was observed in the provider serving populations with higher average SEL. These findings reflect sector-level differences rather than individual-level socioeconomic characteristics. Although there are few studies evaluating this relationship, a study in the United States analyzed the use of antipsychotics (number of prescriptions) over a 6-year period, depending on whether the children aged from 1 to 19 years were treated by a public health provider (Medicaid) or through private insurance. A higher prevalence of antipsychotic use (and used for off-label indications) was found (Patel et al., 2005). One of the authors’ hypotheses is that the higher utilization of psychotropic drugs may be related to the fact that the frequency of psychiatric conditions is higher and their severity more serious among patients in the public health subsector (Medicaid). It was in this sector where less access to psychotherapy was evidenced (Patel et al., 2005). These findings may reflect a greater reliance on pharmacological treatment strategies within healthcare sectors serving populations with lower average socioeconomic profiles. These patterns may indicate differences in therapeutic approaches across healthcare sectors, particularly in the management of male adolescents, although individual-level socioeconomic characteristics and clinical decision-making processes were not assessed in this study.

In all three SELs was observed in year 2020 an increase, coinciding temporally with the onset of the COVID-19 pandemic. The increase in DDD per 1000 inhabitants/day in adolescents from both low and high socioeconomic status in 2020 could be related to studies that have reported increases in mental health-related visits among children and adolescents during the COVID-19 period (Antoniou et al., 2023; Saunders et al., 2022). It has been observed that the use of psychotropic drugs, especially antidepressants and antipsychotics, increased, in an attempt to treat a range of emotional symptoms as well as mental health illnesses that were considered to be aggravated by the pandemic. Some authors have suggested that these changes may have been related to disruptions in face-to-face services and school attendance during that period (Antoniou et al., 2023).

A population-based study in Canada evaluated the dispensing of antidepressants and antipsychotics in adolescents under 18 years of age in the period 2014 to 2022 and observed an increase in antipsychotic dispensing, immediately beginning in June 2020 when the “face-to-face cutoff” was defined and remained elevated over 2 years of study follow-up (Antoniou et al., 2023). During 2021 and 2022, the health restrictions imposed because of COVID were fewer than in 2020.

Multiple studies have explored the link between the measures adopted for the mitigation of the COVID-19 pandemic and the mental health of adolescents, associated with several factors including, but not limited to, the closure of schools, the interruption of face-to-face contact with friends, worries for one’s own health and that of loved ones, concern for the pandemic-related economic situation, and changes in sleep routines. Some of the impacts attributed to the pandemic reported include post-traumatic stress disorder, symptoms of depression and anxiety, irritability, apathy, feelings of loneliness, decreased life satisfaction, suicidal ideation, self-injury and eating disorders (Galiano et al., 2020; Broche et al., 2020; Magson et al., 2021; Loades et al., 2020). Although our study did not address the rationales for using antipsychotic medication in Uruguay, a report by the Scientific Advisory Group during the pandemic mentions a high prevalence of mental health disorders among children and adolescents during this period, which may have to do with the rises in prescriptions from 2020 onwards (Cabral et al., 2025b).

The most commonly dispensed antipsychotics: risperidone, aripiprazole are consistent with international and national studies (Mastroianni et al., 2017; Dörks et al., 2023; Bushnell et al., 2021; Ismael et al., 2010). In recent years, professionals are increasingly prescribing atypical antipsychotics on the grounds of their better safety profile; they are reported to induce less neurological side effects, such as extrapyramidal effects; however, these drugs have been reported to have metabolic adverse effects (Sánchez and Hervías, 2018; Coustals et al., 2021). The choice of the atypical antipsychotic may vary according to the recommendations of each country or institution, prescription profiles or availability of drugs in healthcare centers.

In the case of pediatrics, aripiprazole became available as an oral solution in Uruguay in 2018; this may explain why the first year of the study (2018) prescriptions were much lower than in the last years. The difference may also be due to the greater availability in the different institutions and to potential changes in prescription preferences in the study period. None of these aspects were collected in the present work. The fact that aripiprazole is the antipsychotic with the best metabolic profile may also make it more popular among prescribers (Coustals et al., 2021).

### 4.2 Limitations

The indicator used in the present study, the DDD per 1,000 inhabitants/day, was developed to evaluate utilization by adults, as a consumption indicator. Therefore its use is a limitation of the study because it is not completely accurate for children. However, and given that there is no DDD for pediatrics and especially for off-label indications, it is maintained as a variable accepted by the WHO for this population, mainly to address comparisons between providers, other regions or other periods in future studies similar to this one.

Some studies have tried to establish a DDD adjusted to children, mainly for antibiotics, comparing theoretical DDD with DDD calculated from prescriptions in hospitalized pediatric patients (Montecatine et al., 2023). It would be interesting to validate DDD for children in other drugs like psychotropic drugs, especially antipsychotics, in order to have an appropriate indicator that will allow us to evaluate the consumption of these drugs.

As mentioned above, the data for describing utilization comes from the dispensing data from pharmacies of each institution as requested, so it does not reflect the real use of the population, although it is the most accepted for calculating the DDD per 1,000 inhabitants/day. Added to the above, one must not forget that this way of measuring based on center dispensing may underestimate the figures, as people may have access through other channels, such as community pharmacies.

This type of methodology is not designed to understand the reason why the treatment was prescribed. The DDD is an estimate of population consumption data and does not accurately reflect the actual indications or usage of individuals. To analyze the reasons for the use of antipsychotics in this population, another type of study should be designed, for example, an indication-prescription study.

Despite these methodological limitations related to estimating psychotropic drug consumption in pediatrics, given the large increase in use in this population, without solid evidence to support it or reliable data on long-term adverse effects, it was deemed necessary to conduct this study in this population.

Another important clarification, although no differences in consumption by geographical area were found in the literature, is that these results mainly represent children and adolescents from the capital of Uruguay and are not applicable to the rest of the country.

Another limitation is the estimate of socioeconomic level using the healthcare providers. Although it has been used in other studies, they do not take individual incomes into account, and instead establish an average among the incomes of all users.

### 4.3 Future directions

It would be interesting to complete this study with data from the whole country and to include data on the indications of these antipsychotics and the duration of treatment, as well as to add qualitative studies to analyze this prescription profile in the health system in greater depth.

In other countries, various strategies are already being implemented that aim to reduce the use of antipsychotics in the pediatric population and promote their rational use, such as promoting greater access to psychotherapy services, sharing clinical decisions with patients, standardizing treatments, designing user monitoring programs, among others (Kelleher et al., 2020). It would be worthwhile to promote the implementation of some of these strategies at the local level.

The factors that determine the differential use at different SEL should be studied further to identify at what stage of the process of mental healthcare it is feasible and to intervene as a priority.

## 5 Conclusion

This is the first study in the country that describes the utilization of antipsychotics in children and adolescents and based on SEL categories. The highest utilization of antipsychotics was observed in the provider classified as serving populations with lower SEL and the lowest utilization in the provider classified as serving populations with higher SEL.

The study showed an increase in the use of antipsychotics among children and adolescents between 1 and 19 years of age in Montevideo in the period 2018 to 2022, with the most dispensed antipsychotics being risperidone, aripiprazole and quetiapine.

The use of antipsychotics was higher among males in all age groups. The highest rates of utilization of antipsychotics were observed in 15- to 19-year-old adolescents, followed by the category schoolchildren-adolescents (5–14 years).

These data, when compared with data obtained using the same methodology, are useful for comparison at the national and international levels, as well as for monitoring changes in usage patterns over time. They can also contribute to public health policy decisions and complement studies that address this topic with different approaches or methodologies.

## Data Availability

The data analyzed in this study is subject to the following licenses/restrictions: The dataset that supports this research is not public; it belongs to each health institution that contributed the data for a global analysis. Requests to access these datasets should be directed to santicabralf@gmail.com.
