# Supplementary material for: Pharmacoepidemiology of antipsychotic utilization among children and adolescents in Montevideo between 2018 and 2022
Source: Front Pharmacol. 2026 Apr 28;17:1778138. doi: 10.3389/fphar.2026.1778138 (PMC13161129; doi:10.3389/fphar.2026.1778138)
Supplement: Supplementary file 1 [file DataSheet1.pdf]

Supplementary tables

“Pharmacoepidemiology of antipsychotic utilization among children and adolescents in Montevideo between 2018 and 2022”

Supplementary table 1. DDD per 1,000 inhabitants/day per drug, sex and age in the period 2018 to 2022 in the Lower Socioeconomic Level.

Drug	2018		2019		2020		2021		2022	
	Male	Female	Male	Female	Male	Female	Male	Female	Male	Female
**1-4 years old**										
Risperidone	0,268	0,069	0,304	0,087	0,212	0,061	0,221	0,066	0,271	0,110
Aripiprazole	0	0	0	0	0,006	0,006	0,006	0	0,020	0
Quetiapine	0	0	0	0	0,002	0	0	0	0,001	0
Olanzapine	0	0	0	0	0	0	0	0,051	0	0
Haloperidol	0	0	0,003	0	0,020	0	0	0	0	0
All antipsychotics*	0,284	0,073	0,355	0,093	0,256	0,069	0,232	0,119	0,314	0,117
**5-14 years old**										
Risperidone	6,513	1,509	6,210	1,484	4,875	1,258	4,847	1,395	5,074	1,557
Aripiprazole	1,680	0,450	2,753	0,738	3,178	0,838	3,845	1,261	4,449	1,606
Quetiapine	0,981	0,268	0,732	0,306	0,852	0,247	0,853	0,214	0,669	0,214
Olanzapine	0,069	0,028	0,233	0,143	0,236	0,414	0,404	0,177	1,173	0,206
Haloperidol	0,421	0,121	0,482	0,185	0,458	0,119	0,377	0,046	0,301	0,034
All antipsychotics*	10,396	2,641	11,278	3,095	10,336	3,091	11,009	3,335	12,255	3,838
**15-19 years old**										
Risperidone	4,702	1,993	4,205	1,711	4,039	1,737	4,343	2,415	5,065	2,960
Aripiprazole	1,017	0,598	1,301	0,878	1,720	1,118	2,471	1,455	3,194	1,925
Quetiapine	2,688	1,344	2,373	1,402	2,317	1,759	2,621	1,935	2,764	2,231
Olanzapine	1,143	0,351	3,330	1,773	3,229	1,234	3,523	1,203	9,429	7,122
Haloperidol	0,924	0,394	1,071	0,163	1,454	0,334	1,183	0,534	0,771	0,340
All antipsychotics*	11,222	4,952	13,210	6,311	13,769	6,591	15,241	7,889	22,630	15,016
**Includes, in addition to the agents listed above, levomepromazine, chlorpromazine, chlorprothixene, lurasidone, periciazine, sulpiride, clozapine and tiapride.*										

Supplementary table 2. DDD per 1,000 inhabitants/day per drug, sex and age in the period 2018 to 2022 in the Middle Socioeconomic Level.

Drug	2018		2019		2020		2021		2022	
	Male	Female	Male	Female	Male	Female	Male	Female	Male	Female
**1-4 years old**										
Risperidone	0,920	0,420	1,215	0,453	1,518	0,283	1,230	0,326	3,804	0,481
Aripiprazole	0	0	0,071	0	0,203	0	0,349	0	0,180	0,022
Quetiapine	0	0	0	0	0	0	0	0	0	0
Olanzapine	0	0	0	0	0	0	0	0	0	0
Haloperidol	0	0,004	0	0	0,003	0	0,010	0	0,196	0
All antipsychotics*	0,959	0,444	1,434	0,510	2,021	0,337	1,766	0,382	4,180	0,505
**5-14 years old**										
Risperidone	7,957	1,472	8,172	1,664	7,309	1,598	6,915	1,742	8,495	2,770
Aripiprazole	2,659	1,592	4,478	1,472	5,686	2,139	7,335	3,100	10,416	4,331
Quetiapine	2,268	0,735	2,511	0,411	1,980	0,447	1,560	0,613	1,754	0,924
Olanzapine	0,019	0,058	0,005	0,013	0	0	0	0	0,023	0,088
Haloperidol	0,724	0,108	0,685	0,506	0,485	0,372	0,278	0,370	0,263	0,148
All antipsychotics*	15,307	4,506	17,827	4,575	18,038	5,220	18,315	6,553	22,811	8,784
**15-19 years old**										
Risperidone	6,236	2,334	8,056	2,538	8,390	3,865	7,810	5,519	7,775	8,265
Aripiprazole	6,583	4,562	9,307	6,928	11,626	10,018	11,096	15,475	14,776	21,818
Quetiapine	5,552	4,888	6,093	4,534	6,374	5,065	5,262	5,434	7,435	7,638
Olanzapine	2,746	0,900	2,323	1,855	1,744	1,432	2,456	1,675	3,249	1,770
Haloperidol	1,606	0,754	1,872	0,153	0,892	0,409	1,093	0,726	1,958	1,190
All antipsychotics*	27,072	14,081	32,865	17,273	34,190	21,827	32,699	30,023	40,906	42,334

**Includes, in addition to the agents listed above, levomepromazine, chlorpromazine, chlorprothixene, lurasidone, periciazine, sulpiride, clozapine and tiapride.*

Supplementary table 3. DDD per 1,000 inhabitants/day per drug, sex and age in the period 2018 to 2022 in the Higher Socioeconomic Level.

Drug	2018		2019		2020		2021		2022	
	Male	Female	Male	Female	Male	Female	Male	Female	Male	Female
**1-4 years old**										
Risperidone	0,241	0,012	0,141	0,039	0,153	0	0,207	0	0,108	0,038
Aripiprazole	0	0	0	0	0	0	0,121	0	0	0
Quetiapine	0	0	0	0	0	0	0	0	0	0
Olanzapine	0	0	0	0	0	0	0	0	0	0
Haloperidol	0	0	0	0	0	0	0	0	0	0
All antipsychotics*	0,281	0,012	0,141	0,044	0,168	0,005	0,348	0	0,108	0,038
**5-14 years old**										
Risperidone	0,776	0,283	0,779	0,258	0,827	0,209	0,709	0,124	0,887	0,141
Aripiprazole	0,036	0,028	0,027	0	0	0,099	0,077	0	0,332	0,026
Quetiapine	0,024	0	0,027	0	0,013	0,007	0,024	0,101	0,056	0,036
Olanzapine	0	0	0	0	0	0	0	0	0	0
Haloperidol	0	0	0,014	0	0,027	0	0	0	0,019	0
All antipsychotics*	1,069	0,371	1,123	0,258	0,988	0,315	0,825	0,263	1,376	0,237
**15-19 years old**										
Risperidone	0,271	0,059	0,166	0,024	0,276	0,471	0,175	0,464	0,222	0,357
Aripiprazole	0	0,178	0	0	0	0	0,125	0	0,164	0
Quetiapine	0,146	0,104	0,223	0,114	0,141	0,188	0,305	0,569	0,825	0,046
Olanzapine	0	0,044	0	0	0	0,147	0	0,238	0	0,089
Haloperidol	0	0	0	0	0,008	0	0,055	0,037	0	0,067
All antipsychotics*	1,048	0,637	1,020	0,212	0,877	0,923	1,370	1,351	0,442	1,376

**Includes, in addition to the agents listed above, levomepromazine, chlorpromazine, chlorprothixene, lurasidone, periciazine, sulpiride, clozapine and tiapride.*
